# Systematic Characterization of High-Power Short-Duration Ablation: Insight From an Advanced Virtual Model

**DOI:** 10.3389/fmedt.2021.747609

**Published:** 2021-11-12

**Authors:** Argyrios Petras, Zoraida Moreno Weidmann, Massimiliano Leoni, Luca Gerardo-Giorda, Jose M. Guerra

**Affiliations:** ^1^Johann Radon Institute for Computational and Applied Mathematics (RICAM), Austrian Academy of Sciences, Linz, Austria; ^2^Department of Cardiology, Hospital de la Santa Creu i Sant Pau, Centro de Investigación Biomédica en Red Enfermedades Cardiovaculares (CIBERCV), Universitat Autónoma de Barcelona, Barcelona, Spain; ^3^Institute for Mathematical Methods in Medicine and Data-Based Modelling, Johannes Kepler University, Linz, Austria

**Keywords:** radiofrequency ablation, high-power short-duration, computational modeling, lesion science, ablation catheter

## Abstract

**Background:** High-power short-duration (HPSD) recently emerged as a new approach to radiofrequency (RF) catheter ablation. However, basic and clinical data supporting its effectiveness and safety is still scarce.

**Objective:** We aim to characterize HPSD with an advanced virtual model, able to assess lesion dimensions and complications in multiple conditions and compare it to standard protocols.

**Methods:** We evaluate, on both atrium and ventricle, three HPSD protocols (70 W/8 s, 80 W/6 s, and 90 W/4 s) through a realistic 3D computational model of power-controlled RF ablation, varying catheter tip design (spherical/cylindrical), contact force (CF), blood flow, and saline irrigation. Lesions are defined by the 50°C isotherm contour. Ablations are deemed safe or complicated by pop (tissue temperature >97°C) or charring (blood temperature >80°C). We compared HPSD with standards protocols (30–40 W/30 s). We analyzed the effect of a second HPSD application.

**Results:** We simulated 432 applications. Most (79%) associated a complication, especially in the atrium. The three HPSD protocols performed similarly in the atrium, while 90 W/4 s appeared the safest in the ventricle. Low irrigation rate led frequently to charring (72%). High-power short-duration lesions were 40–60% shallower and smaller in volume compared to standards, although featuring similar width. A second HPSD application increased lesions to a size comparable to standards.

**Conclusion:** High-power short-duration lesions are smaller in volume and more superficial than standards but comparable in width, which can be advantageous in the atrium. A second application can produce lesions similar to standards in a shorter time. Despite its narrow safety margin, HPSD seems a valuable new clinical approach.

## Introduction

Radiofrequency (RF) catheter ablation is the cornerstone treatment for cardiac arrhythmias. Radiofrequency lesions depend mainly on the power delivered to the tissue, catheter stability, and contact force (CF) over time (1, 2). In the initial development of RF ablation in the 90's, a power titration was performed according to efficacy and safety targets (temperature registered by the catheter, impedance drop, signal analysis), between 20 and 50 W. Clinical experience and extensive research in the field, aiming at maximizing the lesion size and minimizing the risk of complications, led to protocols where the power and duration have been standardized according to the arrhythmogenic substrate. For 4 mm-tip catheter the general consensus is to limit the maximum power to 30–50 W over 30–90 s (2, 3). Nevertheless, catheter stability and optimal CF over time are challenging to keep in a beating heart. This may result in smaller and inconsistent lesions that hamper the creation of effective lines of electrical block and prolong the duration of the procedures.

Aimed at overcoming these limitations of conventional protocols, a new RF ablation paradigm has emerged in the last years. Known as high-power short-duration (HPSD) ablation, these protocols are based on increasing maximum power and markedly shortening the application. A heterogeneity of power/duration settings, from 35 to 60 W and 6 to 15 s, have been proposed and tested for pulmonary vein isolation (4). Some groups have investigated more aggressive protocols with power ranging from 70 to 90 W during 4–8 s (5, 6). However, lesion science of HPSD is largely unknown and the results of *in-vivo* and *in-vitro* studies comparing these lesions to standard ones are contradictory (6–8). Moreover, clinical data supporting their effectiveness and safety is limited (9, 10). Additionally, as most of the clinical studies are on pulmonary vein isolation, experience on other atrial substrates is limited and absent on the ventricle.

The development of advanced virtual models for RF ablation allows to simulate lesion formation mimicking infinite scenarios. A systematic and realistic evaluation of RF ablation can be performed by changing parameters as catheter type and shape, ablation protocol, blood and irrigation flow, CF, or cardiac chamber. This will grant an understanding of RF lesion formation and its complications beyond that obtained through classical *in-vitro* and *in-vivo* assessment. This novel research approach proves of particular interest in early stages of new ablation strategies like HPSD.

We aim to systematically characterize different HPSD ablation protocols by means of an advanced virtual model developed by our group (11). Lesion dimensions and complications will be evaluated and compared to those of standard conventional protocols, providing valuable insight in view of the clinical application of HPSD.

## Methods

### Virtual Model

Our computational model of RF catheter ablation simulates a human heart considering fixed conditions, to standardize the comparison of the different ablation settings. The mathematical model, based on an *in-vitro* experimental setup (12), is built within a three-dimensional computational framework described previously by our group (11). It considers tissue elasticity, thermoelectric interaction between electrode and ablation area, and convective cooling by blood flow and irrigation saline. In Petras et al. (11) the model has also been validated against *in-vitro* experimental data for standard RF protocols. A detailed description of the model is given in the Supplementary Material.

The biophysical properties of the tissue introduced in the model are tissue density, thermal conductivity and heat capacity (Supplementary Table 1 of the Supplementary Material), whose values are based on experimental data registered in IT'IS database (13). Heat expansion through the tissue occurs in a radial manner. We assume a linear temperature-dependent decrease of specific heat (0.42 per 1°C increase) and thermal conductivity (0.05% per 1°C increase), and a linear temperature-dependent increase in electrical conductivity (0.15% per 1°C increase), following the experimental studies of Haines and Watson (14) and Rossmanna and Haemmerich (15). Tissue heterogeneity may influence the biophysical properties, however, due to the lack of data in this respect, we neglected this feature in the model. The governing equation for the thermal problem is the Bioheat Equation, while the blood flow and its interaction with the irrigation saline in the blood compartment are modeled using the incompressible Navier-Stokes equations for fluid dynamics (16). The model is solved by finite elements through our self-developed software in FEniCS-HPC (www.fenics-hpc.org). The computational geometry features about 5,000,000 tetrahedra with a maximum element size of 1 mm and a minimum of 0.01 mm in the area of the electrode, where higher computational accuracy is needed. Simulations were carried out on the cluster Hipatia of BCAM (the Basque Center for Applied Mathematics, Bilbao, Spain) which features 18 nodes (1 with Nvidia Tesla K40 GPU) for 624 cores with 4 TB RAM and Infiniband network connectivity.

### Electrode Tip Design

**Figure 1:**
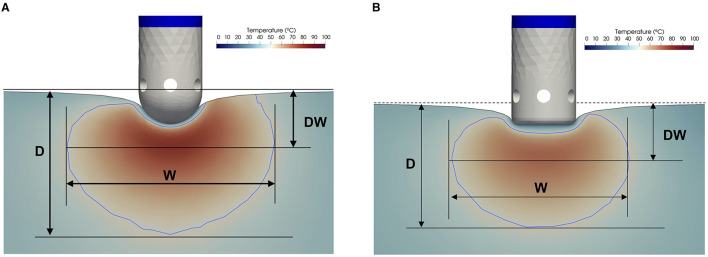
Virtual lesion created with the two considered catheters. **(A)** Spherical tip and **(B)** Cylindrical tip. The area of irreversible tissue lesion (tissue temperature ≥50°C) is delimited by the blue line. Measurements: maximum depth (D), depth at the maximum diameter (DW), maximum diameter (W).

We consider two open-irrigated electrode designs used in clinical practice, and also used in a previous experimental study performed by our group (12), one with a hemispherical tip and one with a cylindrical tip (7 French, 3.5 mm length). Both feature six irrigation pores (0.5 mm diameter) (Figure 1).

### Lesion Assessment

At 50°C, cardiomyocytes exhibit a permanent loss of excitability, as well as a thermally induced necrosis leading to irreversible myocardial injury, which is the target of RF ablation. In light of the above consideration, virtual lesions are identified by the isotherm contour of 50°C within the cardiac tissue, considering irreversible tissue damage at 50°C (17, 18). The assessment of the lesion dimensions is performed with ParaView (19). We measure lesion volume, maximum width (W), maximum depth (D), and depth at the maximum width (DW) (Figure 1), considering the zero at the undeformed endocardial surface.

### Complications Assessment

A steam pop is considered to occur when tissue temperature ≥97°C. We use a temperature threshold <100°C to account for the natural fluctuation of water boiling point depending on tissue composition and other physical conditions (20). Although reaching a boiling temperature doesn't automatically result in pop, given the seriousness of this complication, we equate the risk of pop with the occurrence of it. Charring is assumed to be initiated at blood temperature of 80°C. The occurrence of one of the two complications ends the application, precluding the development of the other. Applications that finish without any complication are deemed safe.

### Ablation Settings

Three different HPSD ablation protocols are used (70 W/8 s, 80 W/6 s, 90 W/4 s) delivered in power-control mode without temperature limit. The model considers a fixed impedance of 120 Ohms, mimicking the behavior of contemporary RF generators, that vary the RF current delivery according to impedance changes, to ensure the delivery of the selected power. Since the chosen power is a fixed parameter, changes in baseline impedance and during ablation are disregarded. Each protocol is simulated on the atrium and the ventricle. All virtual applications are performed perpendicularly to the endocardium. Based on clinical data, we assume similar biophysical properties for both cardiac regions, only differenced by thickness (6 mm in atrium, 12 mm in ventricle) (21). Four increasing CF from 5 to 20 g are simulated resulting in progressive deformation of the tissue.

In order to simulate representative cardiac regions (22) three blood flow velocities are used: no-blood flow (0 m/s), as expected in epicardium or below a valve leaflet; low-blood flow (0.1 m/s), as expected in healthy atrium or pulmonary veins; and high-blood flow (0.5 m/s), as expected at the cavo-tricuspid annulus, ventricle, or outflow tracts.

Additionally, three different saline catheter irrigation rates, are simulated for each HPSD protocol: normal, high, and very high rate (17, 30, and 60 ml/min, respectively, according to the technical specifications of the commercial catheters).

All simulated combinations are summarized in Table 1.

**Table 1:** Summary of the different ablation settings and physiological scenarios considered combining catheter tip, contact force, power, duration, irrigation rate, blood flow, and cardiac chamber.

Ablation settings	Catheter tip	Spherical Cylindrical
	Contact force	5 g 10 g 15 g 20 g
	Power/Duration	70 W/8 s 80 W/6 s 90 W/4 s
	Irrigation rate	17 ml/min 30 ml/min 60 ml/min
Physiological scenarios	Blood-flow	0.0 m/s 0.1 m/s 0.5 m/s
	Ablation site	Atrium Ventricle

### Design of the Study

The study is structured in three steps:

A. We evaluate the safety of the different HPSD ablation settings and protocols.
B. We assess the efficacy of HPSD protocols identified as the safest in the previous step. For both chambers, we compute HPSD lesion sizes and compare them with those generated by standard applications according to the current standard-of-care (30 W/30 s for the atrium and 30–40 W/30 s for the ventricle, CF 10 g, irrigation rate 17 ml/min) (3, 23).
C. We analyze the impact of a second HPSD application on top of the first one. We evaluate changes in terms of safety and lesion size at increasing time intervals (2, 4, 6, 8, and 10 s) between the first and second application. Irrigation rate is kept at 2 ml/min between the two applications, while the protocol irrigation rate is restored 1 s prior to the second application. Biophysical tissue characteristics for the second application account for the changes in thermal and electrical conductivity resulting from the first application.

For B and C, we simulate a low-blood flow (0.1 m/s) for atrium and ventricle, mimicking in the latter the less favorable conditions for ablation and prone for complications, as those that can be found in areas between papillary muscles and cardiac wall.

## Results

A total of 432 RF ablation lesions are simulated by applying the different predefined ablation parameters (catheter tip design, CF, power, duration, saline irrigation rate) and physiological scenarios (blood flow, ablation site).

### Safety of HPSD

**Figure 2:**
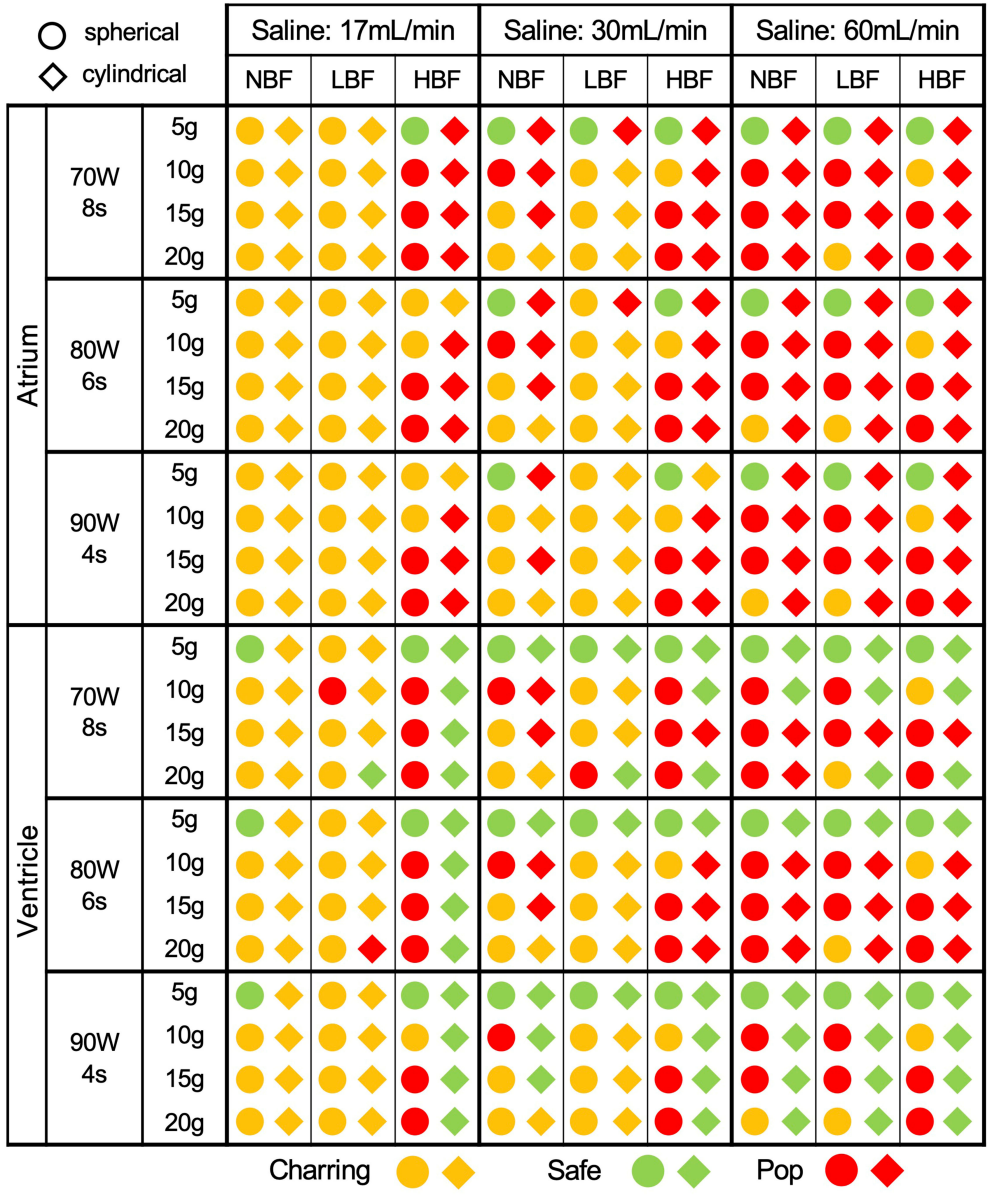
Safety outcomes of HPSD protocols according to the different parameters in Table 1. HBF, high blood-flow (0.5 m/s); LBF, low blood-flow (0.1 m/s); NBF, no blood-flow (0.0 m/s).

**Figure 3:**
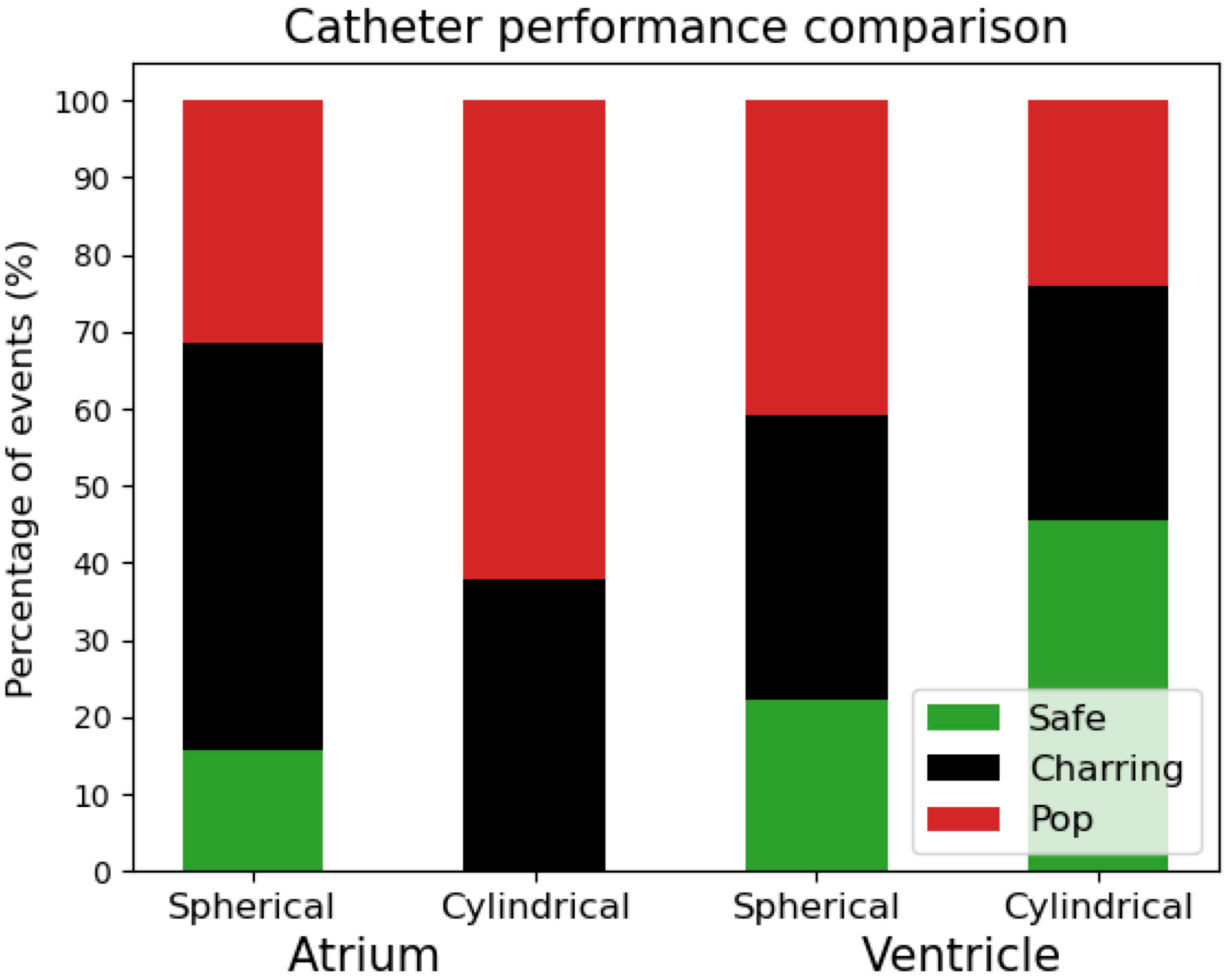
Overall safety comparison of the two different catheter tip designs.

The main findings are summarized in Figure 2 and Supplementary Table 1. Five conclusions can be highlighted:

a. The safety margin of HPSD ablation is, in general, tight, considering that several of the applications associate some complications [342/432 (79%)]. This finding is more notable in the atrium than in the ventricle [199/216 (92%) vs. 143/216 (66%)].
b. Limited electrode tip cooling, through saline flow and/or blood flow, mostly leads to charring: at 17 ml/min, 94% of applications with no-blood flow (45/48) lead to charring in contrast with 15% with high-blood flow (7/48). Charring largely appears at the very beginning of the application (<1 s). Increasing the flushing rate avoids charring and allows to continue the application, which is however often interrupted shortly before the end because of the appearance of pop, as it is the case in 67% of applications at 60 ml/min and no-blood flow (32/48).
c. At low CF, all HPSD protocols exhibit low incidence of pop. In the specific, pops occur in 31% of applications in the atrium (17/54), and never (0/54) in the ventricle. Higher CF result safe only in the ventricle, where 41% applications (66/162) are free of pop for CF >5 g.
d. Different catheter tip designs show varied behaviors (Figure 3). Spherical tips appear relatively safe in both chambers, while cylindrical tips only when used in the ventricle. Nevertheless, cylindrical tips in the ventricle allow the use of larger CF.
e. The complication distribution (charring vs. pop) is similar for both catheters in the ventricle, whereas in the atrium the spherical tip produces more charring and the cylindrical more pops (Figure 3).

### Efficacy of HPSD

In order to assess the efficacy of HPSD protocols, we considered the safest setting emerged from the previous set. These are identified from Figure 2 as the configurations that ensure a safe termination for all protocols. These settings are represented by CF 5 g, irrigation rate 60 ml/min, using a spherical tip in the atrium and a cylindrical tip in the ventricle.

**Figure 4:**
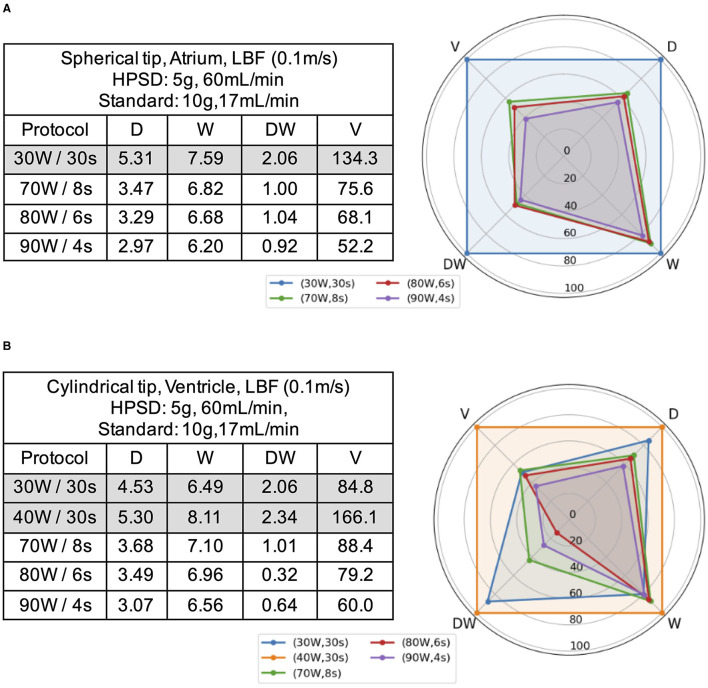
Comparison between HPSD and standard ablation protocols in atrium **(A)** and ventricle **(B)**. Radar charts show the percentage comparison against a reference standard protocol (30 W/30 s in the atrium, 40 W/30 s in the ventricle). The associated tables present the absolute values. D, lesion depth; DW, Depth at maximum width; V, volume; W, lesion width.

The analysis of the different HPSD protocols shows that as power increases and duration diminishes, lesions tend to be narrower and shallower, resulting in a smaller volume (Figure 4). High-power short-duration generates lesions that are always more superficial than those obtained with standard protocols but comparable in width. In the atrium HPSD lesions are always smaller in volume than those obtained with standard protocols, while in the ventricle HPSD lesions are smaller when compared to the ones of the 40 W/30 s protocol, but similar to the 30 W/30 s standard ones (with the exception of the 90 W/4 s protocol whose lesions were consistently of smaller volume).

**Figure 5:**
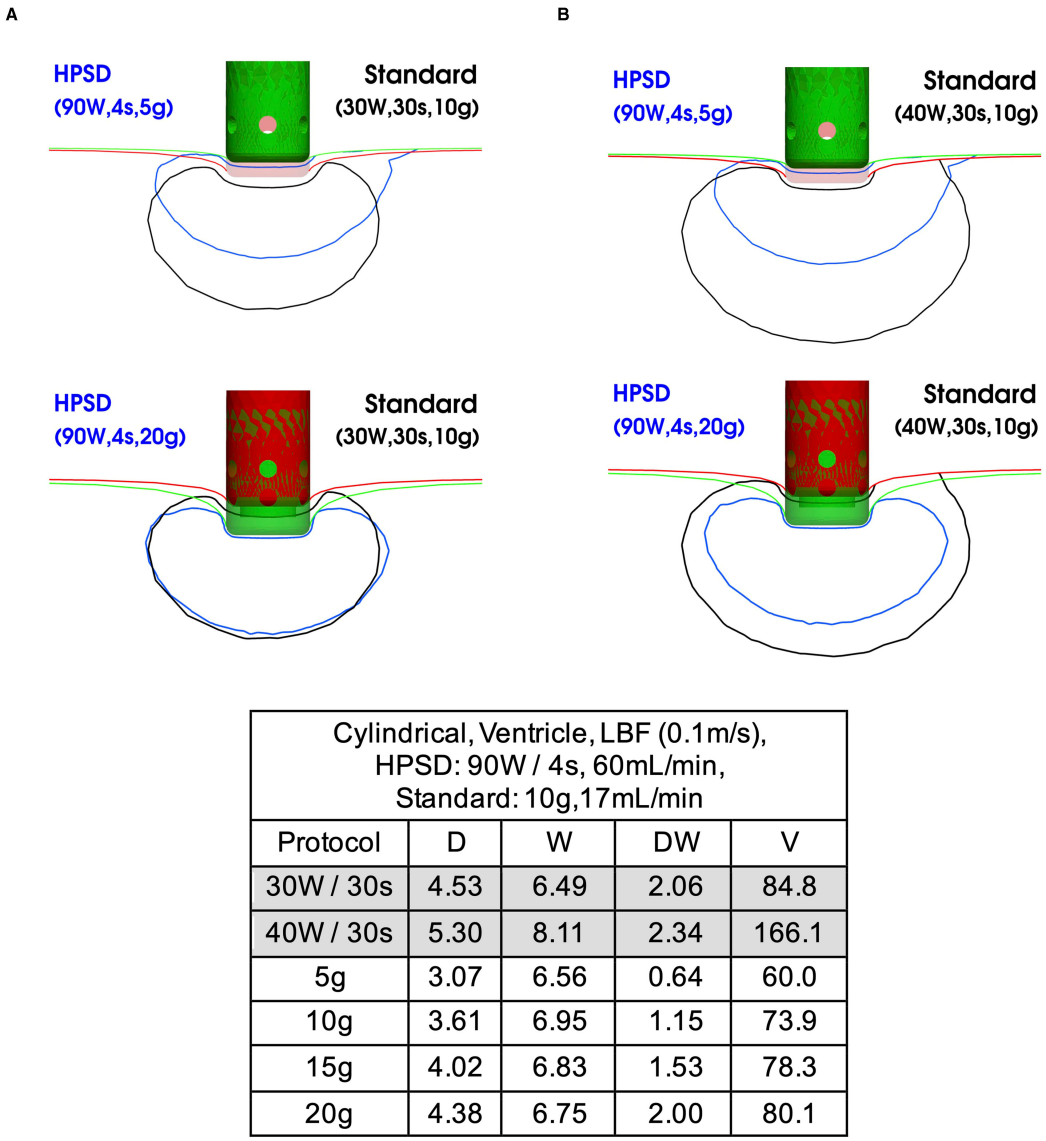
Lesion comparison between 90 W/4 s and standard protocols in the ventricle at different CF. The table collects the absolute values. **(A)** Comparison of 90 W/4 s vs. 30 W/30 s protocol at CF 5 and 20 g; **(B)** comparison of 90 W/4 s vs. 40 W/30 s protocol at CF 5 and 20 g.

Additionally, we analyze the effect of increasing CF in the ventricle for the 90 W/4 s protocol with a cylindrical catheter which was the only HPSD setting that allowed safe applications at higher CF. At CF 20 g, lesions become comparable to the ones from standard 30 W/30 s protocols but remain markedly smaller in volume than those from 40 W/30 s protocols (Figure 5; Supplementary Figure 1).

### Repeated HPSD Applications

**Figure 6:**
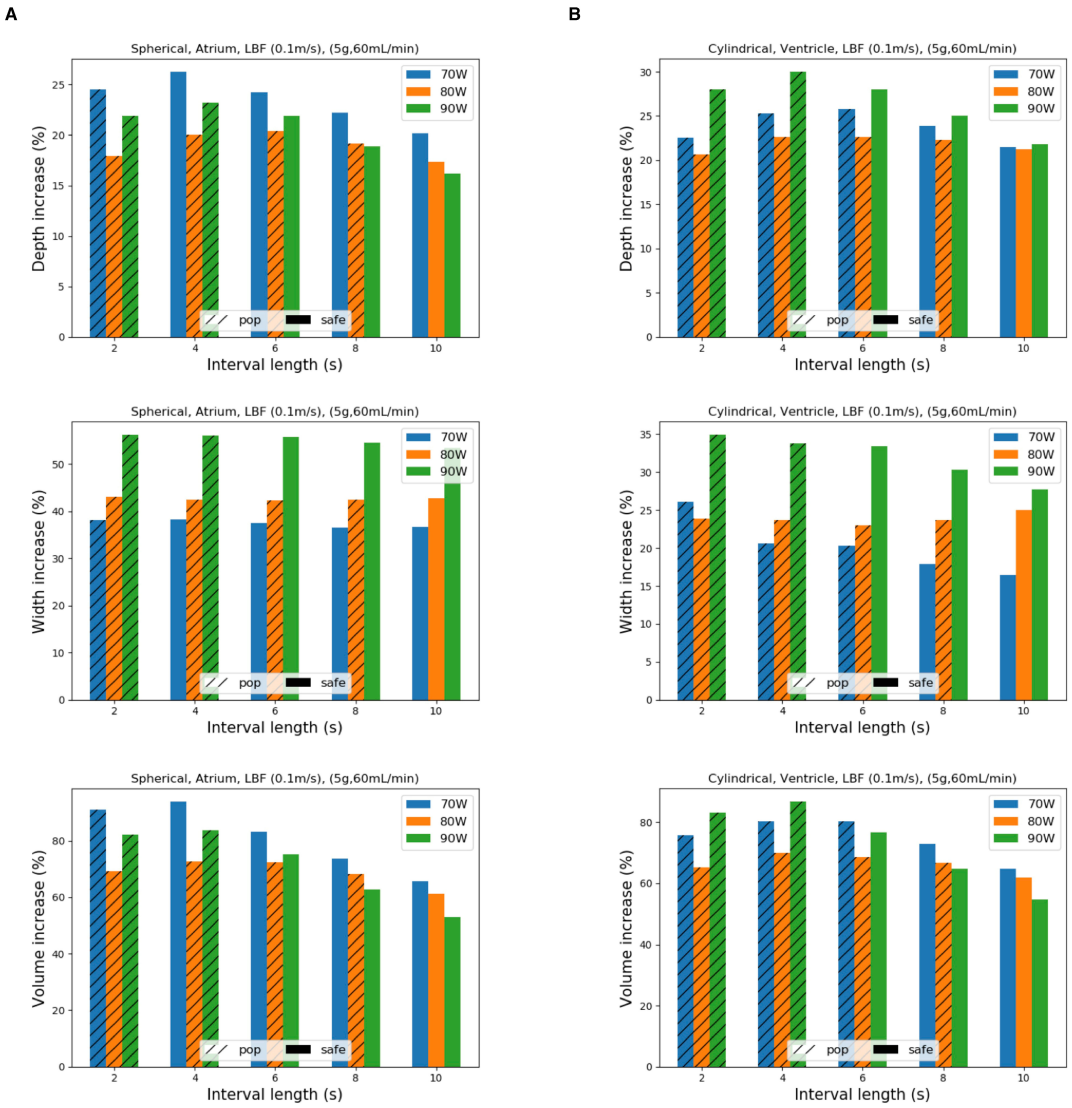
Effect on lesion size from a second HPSD application on top of a first one as a function of the time interval between the two. Ablations are performed with spherical tip in the atrium and cylindrical tip in the ventricle. The percentage increase in depth, width and volume is represented as bar charts for atrium **(A)** and ventricle **(B)**. Striped bars indicate a pop occurrence during the second application.

A second identical HPSD application on top of a first one is simulated after a variable time interval. We consider a spherical tip in the atrium and a cylindrical tip in the ventricle, CF 5 g, low-blood flow and irrigation rate of 60 ml/min, in accordance with the results of the first step. In general, all lesions increase in size after a repeated application (Figure 6; Supplementary Table 2). As the time interval between applications gets longer, lesion size increase is reduced. Furthermore, as the interval decreases, the risk of pop appearance is higher. For both cardiac chambers, the 80 W/6 s protocol shows the worst profile, requiring the longest interval between both applications to avoid complications.

In the atrium, increase in width is more pronounced than in depth, being more evident at 90 W/4 s. Only 70 W/8 s applications with a 4-s interval reach a final lesion volume that exceeds that of a lesion performed with a standard protocol (Supplementary Figure 2).

In the ventricle, the lesion increases similarly in depth and width. The best trade-off between safety and lesion increase is reached with a 90 W/4 s protocol and 6-s interval (showing a 77% volume increase). The final lesion after a second application is larger than the standard 30 W/30 s lesion for any HPSD protocol, especially in terms of width, but generally of smaller volume when compared to those obtained with 40 W/30 s (Supplementary Figure 4).

## Discussion

This is the first study where an advanced virtual model is used to systematically evaluate HPSD ablation in atrium and ventricle. In addition, our innovative model provides a comprehensive insight in the mechanisms of RF ablation, setting the basis for future research in the field. The main advantage of a computer model is that it enables a controlled introduction of a wide range of parameters that influence lesion formation and produces direct results. It circumvents the need for extensive statistical analysis required by *in-vivo* and *in-vitro* experimentation where such parameters are not always under control.

The major findings of the study are: 1. The safety margin of HPSD is in general tight; 2. Catheter tip design and irrigation rate are critical to ensure safe and efficient applications; 3. High-power short-duration lesions are in general shallower and smaller in volume compared to standard lesions; 4. A second HPSD application after a suitable time lag increases lesion size.

### Model Validation

Our model has been previously validated with standard *in-vitro* ablation protocols of porcine tissue (11). For this setting, we have shown that our lesions correlate well in depth but have a tendency to slightly underestimate width. This is likely related to an anisotropic electrical/thermal conduction in the tissue. Nevertheless, since this anisotropic conduction has never been evaluated neither *in-vitro* nor *in-vivo*, we had to use an isotropic conduction for our model.

The study of Kotadia et al. (24) collects experimental data on a variety of tissues, featuring several different protocols, both standard and HPSD. Unfortunately, several details such as CF and saline irrigation for the studies considered in their review are incomplete. Moreover, as we observed in a previous study, the interspecies variability in biophysical properties results in non-negligible differences in the lesion size (25). Hence, a direct validation of our HPSD result is not really feasible. Nevertheless, we can observe (Table 2) an adequate correlation with depth and a slight underestimation of width, for both HPSD and standard protocols. Such consistency in width underestimation does not hamper a fair comparison between our results for standard and HPSD protocols.

**Table 2:** Comparison between HPSD and standard ablation protocols derived from several *in-silico, ex-vivo*, and *in-vivo* studies compiled by Kotadia et al. (24) against the lesions simulated by our model in atrium and ventricle.

**Protocol**	**Energy**	**Depth**			**Width**		
**(W/s)**	**(J)**	**(mm)**			**(mm)**		
		**Kotadia et al**.	**Virtual lesion**		**Kotadia et al**.	**Virtual lesion**	
			**Atrium**	**Ventricle**		**Atrium**	**Ventricle**
30 W/30 s	900	5.1–5.7	5.31	4.53	7.9–8.9	7.59	6.49
70 W/8 s	560	4.0–4.3	3.47	3.68	10.5–10.8	6.82	7.10
80 W/6 s	480	2.9–3.9	3.29	3.49	7.9–11.2	6.96	6.96
90 W/4 s	369	2.6–3.6	2.97	3.07	5.9–10.3	6.20	6.56

*For that purpose, the power/s protocols are converted to total energy delivery. D, lesion depth; W, lesion width*.

### Safety Margin of HPSD

The experience with standard RF protocols is wide and the currently used have demonstrated to be mostly safe (2, 3). High-power short-duration represents a new approach to RF ablation aimed at reducing ablation time and overcoming some limitations of standard protocols. In agreement with the published data (9, 10), our results show that HPSD can be effectively used, although its safety margin is narrow. According to our study, the main determinants of complications are the irrigation rate and the catheter tip design, the latter mostly related to the chamber where RF is applied. To the best of our knowledge, this is the first time that an association between catheter tip shape, anatomical location of ablation and safety is shown. These findings suggest that HPSD should be clinically used with caution, additional technological safety tools should be implemented in the catheters and generators, and operators should use the technology strictly following industry recommendations ensuring stable position and adequate CF.

### Influence of Catheter Tip Design

Catheter tip shape has a crucial impact on the safety profile of HPSD. We observed that while a spherical catheter tip provides the best trade-off between safety and efficacy in the atrium, the opposite occurs in the ventricle.

A possible explanation can be found in the physics of RF ablation. The power delivered to the tissue increases proportionally to the percentage of the catheter tip that is in contact with the endocardium (1), which is different for each catheter according to the CF. At low CF, the entire base of the cylindrical tip is in contact with the tissue, while only a tiny portion of the spherical tip does so. Increasing the CF, the tissue progressively deforms, and the contact surface increases with the pressure. This increase is more pronounced for the spherical tip. As a consequence, at low CF (<10 g) spherical tips feature a smaller contact surface between tissue and electrode that cylindrical tips, while as the CF increases (>10 g) this relationship is reversed (Supplementary Figure 3). As a result, at low CF the spherical tip delivers less power than the cylindrical one while at high CF the opposite occurs. Heat dissipation in the thinner atrial wall is lower (14), limiting the power that can be safely delivered to this chamber. In this case, only the spherical tip at low CF appears to remain within the safety margins. Contrarily, the thicker ventricular wall allows higher power delivery, and the cylindrical tip appears more effective and safer.

Although few studies have analyzed the role of electrode tip shape on standard ablations, a similar pattern was already observed with standard protocols in previous *in-vitro* studies (12) comparing two commercially available catheters, ThermoCool® (Biosense Webster, cylindrical design) and CoolPath® (St Jude Medical, spherical design).

### Catheter Tip Design and Effective Cooling

As previously demonstrated (26), the use of catheter cooling systems allows larger power delivery preventing charring, particularly in areas with poor blood flow. Based on our results, a considerably high flushing rate (>30 ml/min) is necessary to avoid charring, which can be a limitation for heart failure patients. The spherical tip also led more frequently to charring, especially in the atrium, possibly related to a larger surface of the tip in contact with blood than with tissue.

Charring mostly appears in our model at the interface between the metallic tip and the rest of the catheter body. We replicate a cooling system based on six distal coplanar pores, with saline flowing out perpendicularly to the electrode surface. This is probably not the most suitable configuration since the electrode-body interface is not directly cooled down. This advocates for a more efficient cooling system, which could be achieved by either adding more pores distributed along the entire metallic tip, or by forcing a directionality in the irrigation flow toward the upper part of the electrode (6, 7).

### HPSD Lesion Size and Shape

In the line of data reported in HPSD *in-vitro* experiments (6, 7, 24), HPSD lesions are wider than they are deep and, in general, smaller in volume than those of standard protocols. This can be expected since the total energy delivered by HPSD protocols is lower than for the standard protocols (e.g., 360 J for a 90 W/4 s vs. 900 J for a 30 W/30 s). However, the relationship between energy delivery and lesion size is not the same for HPSD and standard protocols. Increases in power, combined with shorter duration, entail a decrease in depth accompanied by minor changes in width. This can be explained by the distinctive biophysics of heat transfer in HPSD protocols, since lesion generation is mainly based on resistive heating, while conductive heating has only a marginal role due to the short duration of the application (14). This feature may prove advantageous in the atrium, allowing to treat the arrhythmogenic substrate in its thin wall avoiding complications in its vicinity. However, it may be insufficient to reach deep targets as in the ventricle or for mitral annular lines (27).

On the contrary, Leshem et al. (6) reported a better linear continuity and transmurality with HPSD ablations than with standard applications. However, this observation could be related to other factors like catheter stability or constant CF during application, more difficult to maintain during standard protocols (6, 8).

### HPSD Protocols Comparison

As power is increased and duration reduced shallower lesions are generated with minimal change in width. In terms of safety, we didn't find any difference between HPSD protocols in the atrium. In contrast, in the ventricle, the 90 W/4 s protocol represented the best power/duration balance to ensure safe lesions while the 80 W/6 s appeared to be the poorest. The 70 W/8 s protocol offered a safe margin only at moderate CF.

Leshem (6) also identified the 90 W/4 s as the best power/duration combination, allowing them to safely apply CF as high as 40 g. Differently to our approach, these authors used a highly sensitive and efficient temperature-controlled catheter with rapid power downregulation at temperature >65°C and a cylindrical tip design with 64-pores, which can explain the wider safety margin with respect to CF.

### Effect of a Second Application

We observed a substantial increase in lesion size after a second identical HPSD application, in opposition to what is expected to occur with standard protocols (28). According to thermodynamics, heat transfer within the tissue continues until thermal equilibrium is reached. This process is estimated to last 45–60 s which is close to the end of application in standard protocols. The short duration of HPSD protocols allows to exploit this thermal latency period by rapidly adding a second application to increase the lesion size.

We analyzed different intervals between applications. The optimal interval was a trade-off between efficacy and safety: the shorter the interval the larger the lesion size gain but, at the same time, the higher the risk of pops.

Two additional findings should be highlighted. In the atrium, the gain in lesion size was more evident in width than in depth, which would potentially maximize the extension of atrial lesions, while preserving neighboring structures. In the ventricle, the second application achieved lesions comparable in size to standard ones, notably reducing the ablation time.

## Limitations

Our advanced virtual model includes some variables which influence the biophysics of RF ablation, making it comparable to experimental models. The model assumes constancy of other variables like catheter orientation, that cannot be fully controlled in clinical practice and would probably change the characteristics of the RF lesion. Due to a lack of data in this respect, we are considering isotropic thermal and electrical conductivities, which produce more spherically-shaped lesions. As the cardiac tissue is not isotropic (fibers orientations, tissue composition), this modeling choice is likely responsible for the lesion width underestimation we observe with respect to some experimental studies. In our opinion, although deserving further investigation, these aspects don't have a significant impact on the global results of this study since they are common limitations for both standard and HPSD protocols. The main objective of the study was to explore the biophysical characteristics of HPSD in comparison to standard RF protocols, therefore extrapolation to the clinical setting should be made cautiously.

Our model assumes a power-controlled system, without upper temperature limit. Adding a self-down-regulation of the energy at a given tissue temperature would probably avoid some complications observed in our simulations, but would prevent a deeper understanding of the behavior of this type of energy delivery.

The simulated applications were interrupted when blood or tissue temperature reached complication thresholds. Our model uses a simple six-pores irrigation system prone to charring formation. The question lingers whether by using new catheters with more effective irrigation system charring could have been avoided and if so, whether pops would have occurred had the applications continued. According to our simulations, enhanced irrigation designs would have little impact on the risk of pop or lesion size.

## Conclusions

High-power short-duration ablation generates shallower and smaller lesions than those of standard protocols. In the atrium, these characteristics can be exploited to reach the arrhythmogenic substrate preserving the neighboring structures. In the ventricle, a second application can create lesions comparable to the ones obtained with standard protocols in shorter time. The safety margin of HPSD is narrow but seems to be a valuable new clinical approach. For best outcomes, it should be implemented cautiously and probably using *ad-hoc* designed RF systems.

## Data Availability Statement

All data supporting the findings of this study are available within the article and its Supplementary Material.

## Author Contributions

JG and LG-G conceived the original idea. LG-G, AP, and ML developed the computational model and performed the simulations. JG and ZMW assessed on the clinical aspects. AP and ZMW drafted the manuscript. All authors discussed the results and contributed to the final manuscript.

## Funding

Part of this work was performed when LG-G, AP, and ML were with the Basque Center for Applied Mathematics (BCAM) in Bilbao, Spain. This research was supported by the Basque Government through the BERC 2018-2021 program, and the Spanish State Research Agency (AEI) through the grant RTI2018-093416-B-I00 MULTIQUANT.

## Conflict of Interest

JG served as consultant for Biosense Webster, Boston Scientific and Abbott, received speaker fees from Boston Scientific and Abbott, and received a research grant from Abbott. The remaining authors declare that the research was conducted in the absence of any commercial or financial relationships that could be construed as a potential conflict of interest.

## Publisher's Note

All claims expressed in this article are solely those of the authors and do not necessarily represent those of their affiliated organizations, or those of the publisher, the editors and the reviewers. Any product that may be evaluated in this article, or claim that may be made by its manufacturer, is not guaranteed or endorsed by the publisher.
