# Supplementary material for: Systematic Characterization of High-Power Short-Duration Ablation: Insight From an Advanced Virtual Model
Source: Front Med Technol. 2021 Nov 12;3:747609. doi: 10.3389/fmedt.2021.747609 (PMC8757782; doi:10.3389/fmedt.2021.747609)

Supplementary Figures:

Systematic characterization of High-Power Short-Duration Ablation: Insight from an advanced virtual model.

Argyrios Petras [1], Zoraida Moreno Weidmann [2], Massimiliano Leoni [1], Jose M. Guerra [2,*], and Luca Gerardo-Giorda [1,3]

[1] RICAM, Austrian Academy of Sciences, Linz, Austria

[2] Department of Cardiology, Hospital de la Santa Creu i Sant Pau, CIBERCV, and Universidad Autonoma de Barcelona, Barcelona, Spain

[3] Institute for Mathematical Methods in Medicine and Data-Based Modelling, Johannes Kepler University, Linz, Austria

Correspondence ∗: jguerra@secardiologia.es

Supplementary Figure 1. Lesion comparison between standard ablation protocol (30W/30s and 40W/30s, CF 10g, 17ml/min) and 90W/4s (60ml/min) at different contact forces (5g, 10g, 15g and 20g) on the ventricle. (A) comparison of 90W/4s against standard 30W/30s protocol; (B) comparison of 90W/4s against standard 40W/30s protocol.

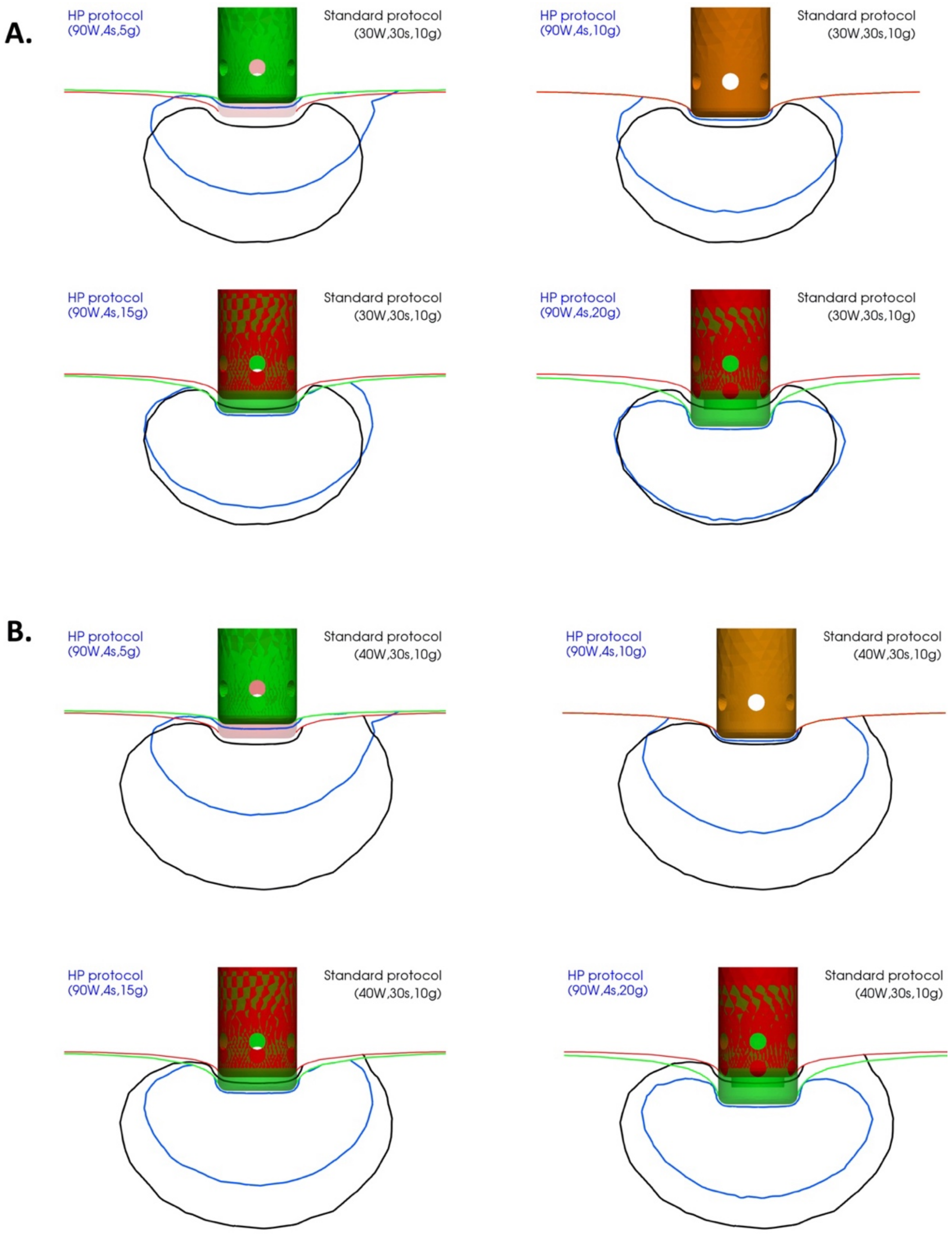

Supplementary figure 2. Graphical representation comparing first HPSD, repeated HPSD and standard ablations. For each HPSD protocol, the interval with the best safe interval/lesion increase balance is represented. First ablation is performed with spherical tip in the atrium and cylindrical tip in the ventricle, at CF 5g, blood flow 0.1m/s and 60ml/min irrigation rate. During the pause a 2ml/min saline inlet is considered and the high saline flow is restored 1s before the second ablation. For the second ablation, the same protocol as the first ablation is considered. For standard protocols, we considered 30W/30s at 5g, 17ml/min in the atrium and 30W/30s and 40W/30s at 10g, 17ml/min in the ventricle. A: atrium; B: ventricle (B1. 90W/4s against 30W/30s and B2. 90W/4s against 40W/30s).

A.

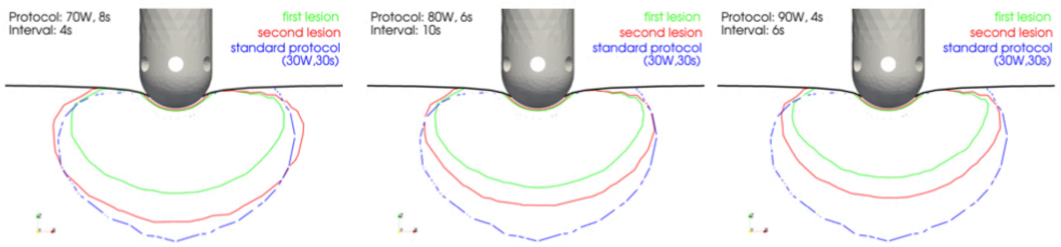

B.

B1.

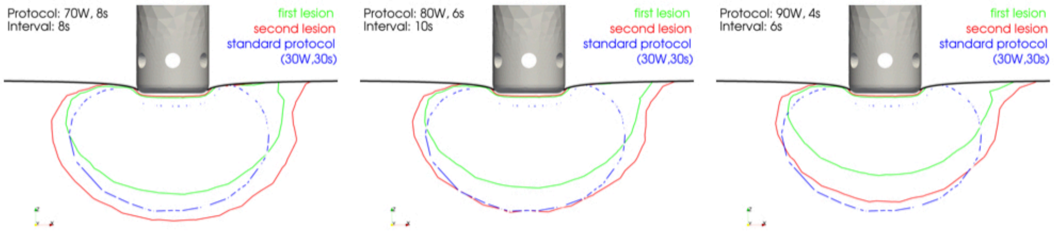

B2.

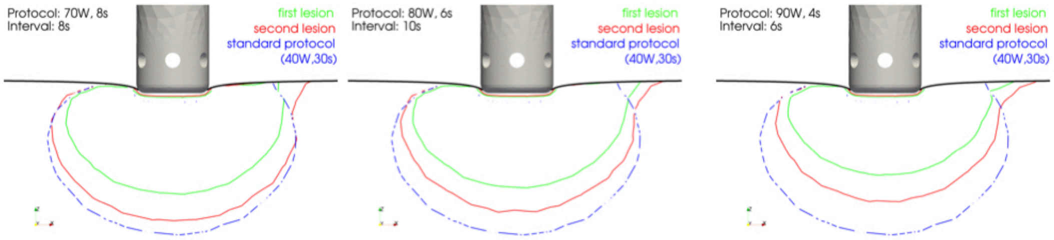

Supplementary figure 3. Influence of the catheter tip design on the percentage of contact with the myocardial tissue for different values of contact force.

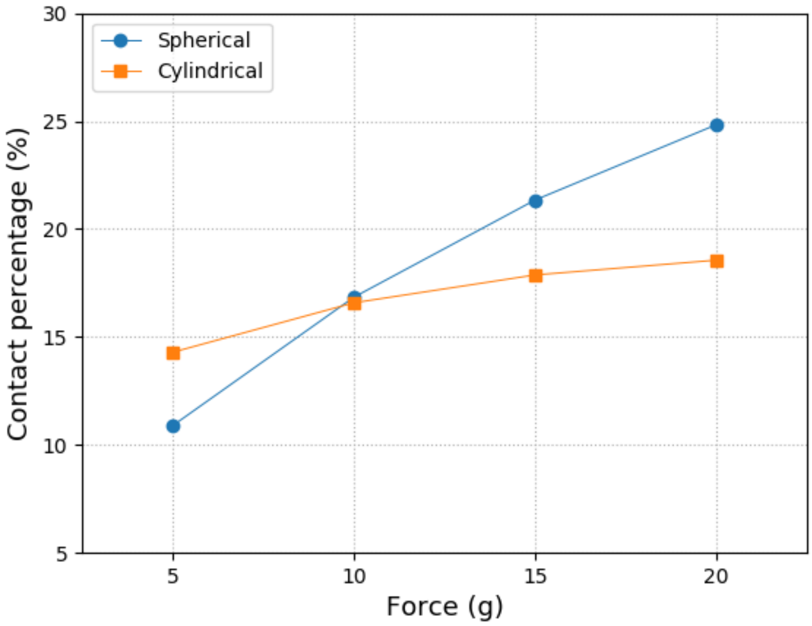

Supplement: Supplementary file 1 [file Data_Sheet_1.PDF]
