# Supplementary material for: Systematic Characterization of High-Power Short-Duration Ablation: Insight From an Advanced Virtual Model
Source: Front Med Technol. 2021 Nov 12;3:747609. doi: 10.3389/fmedt.2021.747609 (PMC8757782; doi:10.3389/fmedt.2021.747609)
Supplement: Supplementary file 2 [file Data_Sheet_2.PDF]

Supplementary Tables:

Systematic characterization of High-Power Short-Duration Ablation: Insight from an advanced virtual model.

Argyrios Petras [1], Zoraida Moreno Weidmann [2], Massimiliano Leoni [1], Jose M. Guerra [2,*], and Luca Gerardo-Giorda [1,3]
[1] RICAM, Austrian Academy of Sciences, Linz, Austria
[2] Department of Cardiology, Hospital de la Santa Creu i Sant Pau, CIBERCV, and Universidad Autonoma de Barcelona, Barcelona, Spain
[3] Institute for Mathematical Methods in Medicine and Data-Based Modelling, Johannes Kepler University, Linz, Austria
Correspondence ∗: jguerra@secardiologia.es

Supplementary tables 1. Safety outcomes of the 432 virtual HPSD ablations performed with both catheter in both chambers modifying the predefined parameters. Values represent: Blood temperature (ºC)/Tissue temperature (ºC)/Time of application (seconds). In green safe lesions, in black lesions resulting in thrombus (blood temperature >80ºC) and in red in pop (tissue temperature >97ºC). Time of application ends if any complication appear. HBF = High Blood Flow (0.5m/s); LBF = Low Blood Flow (0.1m/s); NBF = Blood Flow (0.0m/s).

Spherical Atrium		Irrigation rate: 17 mL/min			Irrigation rate : 30 mL/min			Irrigation rate : 60 mL/min		
		NBF	LBF	HBF	NBF	LBF	HBF	NBF	LBF	HBF
70W 8s	5g	80.0 / 81.6 / 5.2	80.0 / 49.0 / 0.3	76.6 / 88.2 / 8.0	72.8 / 89.7 / 8.0	76.6 / 89.8 / 8.0	72.7 / 88.5 / 8.0	59.9 / 88.7 / 8.0	61.7 / 88.6 / 8.0	66.2 / 88.6 / 8.0
	10g	80.0 / 52.4 / 0.4	80.0 / 48.0 / 0.2	78.8 / 97.1 / 4.3	75.6 / 97.1 / 4.1	80.0 / 53.1 / 0.2	80.0 / 91.5 / 3.4	68.9 / 97.1 / 4.1	66.3 / 97.1 / 4.2	80.0 / 80.0 / 2.0
	15g	80.0 / 45.4 / 0.1	80.0 / 49.1 / 0.2	65.8 / 97.1 / 2.9	80.0 / 49.2 / 0.2	80.0 / 49.2 / 0.2	61.8 / 97.1 / 2.9	59.8 / 97.1 / 2.8	55.1 / 97.1 / 2.8	56.0 / 97.1 / 2.8
	20g	80.0 / 45.8 / 0.1	80.0 / 50.5 / 0.2	59.9 / 97.1 / 2.5	80.0 / 45.8 / 0.1	80.0 / 50.5 / 0.2	58.9 / 97.1 / 2.4	76.0 / 97.1 / 2.4	80.0 / 45.8 / 0.1	60.5 / 97.1 / 2.4
80W 6s	5g	80.0 / 68.6 / 1.8	80.0 / 48.3 / 0.2	80.0 / 88.5 / 5.4	76.9 / 92.1 / 6.0	80.0 / 89.0 / 5.2	75.5 / 91.2 / 6.0	62.6 / 91.5 / 6.0	64.6 / 91.4 / 6.0	71.9 / 91.4 / 6.0
	10g	80.0 / 52.6 / 0.3	80.0 / 49.8 / 0.2	80.0 / 94.3 / 2.8	79.4 / 97.1 / 3.0	80.0 / 55.2 / 0.2	80.0 / 89.0 / 2.3	71.6 / 97.1 / 3.0	68.8 / 97.1 / 3.0	80.0 / 80.0 / 0.8
	15g	80.0 / 46.6 / 0.1	80.0 / 46.6 / 0.1	67.0 / 97.1 / 2.2	80.0 / 51.2 / 0.2	80.0 / 46.2 / 0.1	62.2 / 97.1 / 2.1	62.6 / 97.1 / 2.1	57.8 / 97.1 / 2.1	58.4 / 97.1 / 2.1
	20g	80.0 / 47.2 / 0.1	80.0 / 47.2 / 0.1	62.3 / 97.1 / 1.8	80.0 / 49.0 / 0.1	80.0 / 47.2 / 0.1	61.5 / 97.1 / 1.8	80.0 / 62.2 / 0.4	80.0 / 47.2 / 0.1	64.2 / 97.1 / 1.8
90W 4s	5g	80.0 / 65.8 / 1.0	80.0 / 49.7 / 0.2	80.0 / 83.4 / 3.2	79.2 / 90.0 / 4.0	80.0 / 82.0 / 2.8	78.1 / 89.1 / 4.0	64.4 / 89.4 / 4.0	66.4 / 89.2 / 4.0	72.4 / 89.4 / 4.0
	10g	80.0 / 51.6 / 0.2	80.0 / 51.7 / 0.2	80.0 / 87.2 / 1.7	80.0 / 84.5 / 1.5	80.0 / 57.2 / 0.2	80.0 / 80.8 / 1.3	74.0 / 97.1 / 2.3	71.3 / 97.1 / 2.3	80.0 / 79.3 / 0.5
	15g	80.0 / 47.8 / 0.1	80.0 / 47.8 / 0.1	71.3 / 97.1 / 1.7	80.0 / 53.2 / 0.2	80.0 / 47.3 / 0.1	68.6 / 97.1 / 1.7	64.9 / 97.1 / 1.6	60.2 / 97.1 / 1.6	60.8 / 97.1 / 1.6
	20g	80.0 / 48.6 / 0.1	80.0 / 48.6 / 0.1	65.2 / 97.1 / 1.5	80.0 / 48.6 / 0.1	80.0 / 48.6 / 0.1	64.7 / 97.1 / 1.5	80.0 / 60.5 / 0.3	80.0 / 48.6 / 0.1	68.1 / 97.1 / 1.4

Cylindrical Atrium		Irrigation rate : 17 mL/min			Irrigation rate : 30 mL/min			Irrigation rate : 60 mL/min		
		NBF	LBF	HBF	NBF	LBF	HBF	NBF	LBF	HBF
70W 8s	5g	80.0 / 73.0 / 1.4	80.0 / 50.7 / 0.3	77.6 / 97.1 / 7.2	73.2 / 97.1 / 6.8	74.8 / 97.1 / 6.8	75.3 / 97.1 / 7.2	62.0 / 97.1 / 7.0	62.5 / 97.1 / 7.1	62.7 / 97.1 / 7.1
	10g	80.0 / 52.2 / 0.4	80.0 / 50.7 / 0.3	73.0 / 97.1 / 5.8	73.5 / 97.1 / 5.5	80.0 / 55.8 / 0.3	71.6 / 97.1 / 5.8	63.3 / 97.1 / 5.7	64.4 / 97.1 / 5.8	71.4 / 97.1 / 5.7
	15g	80.0 / 46.5 / 0.2	80.0 / 49.0 / 0.3	65.9 / 97.1 / 6.0	70.4 / 97.1 / 5.7	80.0 / 46.6 / 0.2	60.4 / 97.1 / 6.0	67.8 / 97.1 / 5.7	66.9 / 97.1 / 5.8	63.4 / 97.1 / 5.9
	20g	80.0 / 46.9 / 0.2	80.0 / 51.3 / 0.4	55.4 / 97.1 / 6.9	80.0 / 46.9 / 0.2	80.0 / 49.2 / 0.3	53.9 / 97.1 / 6.8	51.4 / 97.1 / 6.7	62.6 / 97.1 / 6.7	51.9 / 97.1 / 6.8
80W 6s	5g	80.0 / 67.6 / 0.8	80.0 / 49.9 / 0.2	80.0 / 95.3 / 4.6	76.7 / 97.1 / 4.8	78.7 / 97.1 / 4.7	78.5 / 97.1 / 5.0	64.7 / 97.1 / 5.0	65.3 / 97.1 / 5.1	72.6 / 97.1 / 4.9
	10g	80.0 / 52.6 / 0.3	80.0 / 49.8 / 0.2	76.9 / 97.1 / 4.1	77.7 / 97.1 / 3.9	80.0 / 53.8 / 0.2	75.3 / 97.1 / 4.0	66.2 / 97.1 / 4.1	66.4 / 97.1 / 4.1	74.8 / 97.1 / 4.1
	15g	80.0 / 48.2 / 0.2	80.0 / 48.2 / 0.2	69.4 / 97.1 / 4.3	74.6 / 97.1 / 4.0	80.0 / 48.3 / 0.2	63.8 / 97.1 / 4.2	71.1 / 97.1 / 4.1	70.2 / 97.1 / 4.1	65.1 / 97.1 / 4.2
	20g	80.0 / 44.8 / 0.1	80.0 / 51.4 / 0.3	58.1 / 97.1 / 4.8	80.0 / 48.6 / 0.2	80.0 / 48.6 / 0.2	56.9 / 97.1 / 4.8	53.0 / 97.1 / 4.6	66.3 / 97.1 / 4.7	54.4 / 97.1 / 4.7
90W 4s	5g	80.0 / 65.8 / 0.6	80.0 / 51.7 / 0.2	80.0 / 88.7 / 2.6	79.9 / 97.1 / 3.6	80.0 / 92.0 / 2.8	80.0 / 95.3 / 3.4	67.2 / 97.1 / 3.8	67.9 / 97.1 / 3.8	74.2 / 97.1 / 3.7
	10g	80.0 / 54.9 / 0.3	80.0 / 51.5 / 0.2	79.8 / 97.1 / 2.9	80.0 / 94.6 / 2.5	80.0 / 55.6 / 0.2	78.8 / 97.1 / 3.0	68.9 / 97.1 / 3.1	68.3 / 97.1 / 3.1	77.9 / 97.1 / 3.1
	15g	80.0 / 49.9 / 0.2	80.0 / 49.9 / 0.2	72.4 / 97.1 / 3.2	78.4 / 97.1 / 3.0	80.0 / 50.0 / 0.2	66.9 / 97.1 / 3.2	73.9 / 97.1 / 3.0	72.9 / 97.1 / 3.0	67.1 / 97.1 / 3.1
	20g	80.0 / 45.8 / 0.1	80.0 / 50.3 / 0.2	60.2 / 97.1 / 3.5	80.0 / 45.8 / 0.1	80.0 / 50.3 / 0.2	58.9 / 97.1 / 3.5	54.6 / 97.1 / 3.4	70.2 / 97.1 / 3.5	56.4 / 97.1 / 3.5

Spherical Ventricle		Irrigation rate : 17 mL/min			Irrigation rate : 30 mL/min			Irrigation rate : 60 mL/min		
		NBF	LBF	HBF	NBF	LBF	HBF	NBF	LBF	HBF
70W 8s	5g	72.2 / 81.7 / 8.0	80.0 / 49.9 / 0.6	69.9 / 80.0 / 8.0	65.8 / 81.3 / 8.0	69.0 / 81.3 / 8.0	65.7 / 80.4 / 8.0	56.0 / 80.6 / 8.0	56.7 / 80.6 / 8.0	60.3 / 80.5 / 8.0
	10g	80.0 / 54.4 / 0.6	75.9 / 97.1 / 5.8	73.5 / 97.1 / 5.8	70.5 / 97.1 / 5.6	80.0 / 55.1 / 0.3	77.9 / 97.1 / 5.7	65.8 / 97.1 / 5.6	63.1 / 97.1 / 5.6	80.0 / 80.4 / 2.2
	15g	80.0 / 47.8 / 0.2	80.0 / 51.1 / 0.3	61.4 / 97.1 / 3.6	80.0 / 54.3 / 0.4	80.0 / 47.9 / 0.2	57.2 / 97.1 / 3.5	55.2 / 97.1 / 3.5	52.2 / 97.1 / 3.5	52.3 / 97.1 / 3.5
	20g	80.0 / 49.3 / 0.2	80.0 / 59.4 / 0.5	55.8 / 97.1 / 2.8	80.0 / 49.3 / 0.2	74.5 / 97.1 / 2.8	54.9 / 97.1 / 2.8	69.0 / 97.1 / 2.7	80.0 / 49.4 / 0.2	56.2 / 97.1 / 2.8
80W 6s	5g	76.4 / 84.0 / 6.0	80.0 / 49.7 / 0.4	73.4 / 82.2 / 6.0	69.2 / 83.4 / 6.0	72.7 / 83.4 / 6.0	69.1 / 82.6 / 6.0	58.4 / 82.9 / 6.0	58.4 / 82.9 / 6.0	59.1 / 82.8 / 6.0
	10g	80.0 / 55.2 / 0.5	80.0 / 50.0 / 0.3	75.8 / 97.1 / 4.1	73.9 / 97.1 / 4.0	80.0 / 57.3 / 0.3	80.0 / 94.8 / 3.7	68.2 / 97.1 / 4.0	65.5 / 97.1 / 4.0	80.0 / 81.0 / 1.3
	15g	80.0 / 49.1 / 0.2	80.0 / 49.6 / 0.2	64.5 / 97.1 / 2.7	80.0 / 53.5 / 0.3	80.0 / 49.7 / 0.2	59.2 / 97.1 / 2.6	57.5 / 97.1 / 2.6	53.5 / 97.1 / 2.6	55.1 / 97.1 / 2.6
	20g	80.0 / 46.4 / 0.1	80.0 / 55.7 / 0.3	58.3 / 97.1 / 2.1	80.0 / 46.4 / 0.1	80.0 / 59.7 / 0.4	57.3 / 97.1 / 2.1	74.1 / 97.1 / 2.1	80.0 / 51.5 / 0.2	59.3 / 97.1 / 2.1
90W 4s	5g	79.9 / 82.0 / 4.0	80.0 / 49.6 / 0.3	74.4 / 80.6 / 4.0	71.3 / 81.5 / 4.0	74.9 / 81.5 / 4.0	70.0 / 81.1 / 4.0	59.9 / 81.3 / 4.0	60.6 / 81.1 / 4.0	64.6 / 81.4 / 4.0
	10g	80.0 / 55.0 / 0.4	80.0 / 49.5 / 0.2	80.0 / 97.0 / 3.1	77.1 / 97.1 / 3.0	80.0 / 54.1 / 0.2	80.0 / 85.1 / 2.0	70.4 / 97.1 / 3.0	67.7 / 97.1 / 3.0	80.0 / 80.4 / 0.7
	15g	80.0 / 46.3 / 0.1	80.0 / 51.4 / 0.2	67.2 / 97.1 / 2.1	80.0 / 55.8 / 0.3	80.0 / 51.5 / 0.2	63.1 / 97.1 / 2.1	59.7 / 97.1 / 2.0	55.4 / 97.1 / 2.0	57.8 / 97.1 / 2.0
	20g	80.0 / 47.6 / 0.1	80.0 / 53.5 / 0.2	60.7 / 97.1 / 1.7	80.0 / 47.7 / 0.1	80.0 / 53.5 / 0.2	59.7 / 97.1 / 1.7	80.0 / 77.7 / 0.8	80.0 / 53.9 / 0.2	62.5 / 97.1 / 1.6

Cylindrical Ventricle		Irrigation rate: 17 mL/min			Irrigation rate : 30 mL/min			Irrigation rate : 60 mL/min		
		NBF	LBF	HBF	NBF	LBF	HBF	NBF	LBF	HBF
70W 8s	5g	80.0 / 89.4 / 7.2	80.0 / 52.7 / 0.6	69.5 / 88.6 / 8.0	66.7 / 90.3 / 8.0	67.6 / 89.9 / 8.0	68.5 / 89.0 / 8.0	57.3 / 89.6 / 8.0	57.6 / 89.2 / 8.0	56.5 / 89.2 / 8.0
	10g	80.0 / 54.1 / 0.7	80.0 / 49.4 / 0.4	67.9 / 95.8 / 8.0	67.7 / 97.1 / 8.0	80.0 / 58.2 / 0.7	66.5 / 96.1 / 8.0	60.4 / 96.7 / 8.0	61.2 / 96.5 / 8.0	67.1 / 96.6 / 8.0
	15g	80.0 / 47.2 / 0.3	80.0 / 52.6 / 0.6	60.9 / 97.1 / 7.9	64.3 / 97.1 / 7.6	80.0 / 47.2 / 0.3	56.0 / 97.1 / 7.8	62.5 / 97.1 / 7.5	61.6 / 97.1 / 7.6	60.5 / 97.1 / 7.7
	20g	80.0 / 45.2 / 0.2	68.5 / 95.7 / 8.0	51.8 / 95.9 / 8.0	80.0 / 47.4 / 0.3	73.1 / 96.1 / 8.0	50.7 / 96.2 / 8.0	49.6 / 97.1 / 8.0	57.5 / 96.9 / 8.0	48.9 / 96.7 / 8.0
80W 6s	5g	80.0 / 81.7 / 3.3	80.0 / 52.0 / 0.4	73.5 / 90.9 / 6.0	70.2 / 92.2 / 6.0	71.5 / 92.1 / 6.0	70.4 / 91.1 / 6.0	59.9 / 91.8 / 6.0	60.2 / 91.0 / 6.0	59.3 / 91.3 / 6.0
	10g	80.0 / 53.3 / 0.5	80.0 / 49.8 / 0.3	72.0 / 97.1 / 5.9	71.7 / 97.1 / 5.7	80.0 / 54.7 / 0.3	70.3 / 97.1 / 5.8	62.7 / 97.1 / 5.7	63.0 / 97.1 / 5.8	70.2 / 97.1 / 5.7
	15g	80.0 / 46.6 / 0.2	80.0 / 49.0 / 0.3	64.6 / 97.1 / 5.6	67.8 / 97.1 / 5.4	80.0 / 49.2 / 0.3	58.9 / 97.1 / 5.6	65.4 / 97.1 / 5.4	64.4 / 97.1 / 5.4	62.8 / 97.1 / 5.5
	20g	80.0 / 46.6 / 0.2	74.0 / 97.1 / 6.0	54.6 / 97.1 / 6.0	80.0 / 46.6 / 0.2	80.0 / 51.5 / 0.4	53.3 / 97.1 / 5.9	50.6 / 97.1 / 5.7	60.5 / 97.1 / 5.8	51.4 / 97.1 / 5.8
90W 4s	5g	80.0 / 73.0 / 1.3	80.0 / 51.3 / 0.3	76.2 / 88.1 / 4.0	72.3 / 89.1 / 4.0	73.9 / 89.4 / 4.0	73.9 / 88.1 / 4.0	61.4 / 88.5 / 4.0	61.7 / 87.8 / 4.0	61.1 / 88.3 / 4.0
	10g	80.0 / 53.5 / 0.4	80.0 / 48.8 / 0.2	74.6 / 94.5 / 4.0	74.9 / 95.8 / 4.0	80.0 / 52.8 / 0.2	73.1 / 94.7 / 4.0	64.8 / 95.0 / 4.0	64.5 / 94.7 / 4.0	72.7 / 94.9 / 4.0
	15g	80.0 / 48.1 / 0.2	80.0 / 51.0 / 0.3	66.6 / 95.6 / 4.0	71.2 / 96.9 / 4.0	80.0 / 48.2 / 0.2	61.8 / 96.0 / 4.0	68.1 / 96.9 / 4.0	67.0 / 96.7 / 4.0	63.7 / 96.4 / 4.0
	20g	80.0 / 48.0 / 0.2	80.0 / 51.0 / 0.3	56.4 / 94.3 / 4.0	80.0 / 48.0 / 0.2	80.0 / 51.1 / 0.3	55.2 / 94.5 / 4.0	51.8 / 95.3 / 4.0	63.7 / 95.1 / 4.0	53.1 / 94.8 / 4.0

Supplementary tables 2. Effect of repeated HPSD ablations after stepwise time lapses. First ablation is performed with spherical tip in the atrium and cylindrical tip in the ventricle, both at CF 5g, blood flow 0.1m/s and 60ml/min irrigation rate. During the pause a 2ml/min saline inlet is considered and the high saline flow is restored 1s before the second ablation. For the second ablation, the same protocol as the first ablation is considered. Gray shaded values are lesion measurements of the first ablation. In red, applications resulting in pop.

Cylindrical, Ventricle, LBF (0.1m/s), (5g,70W,8s,60mL/min)							
Interval	D*	W	DW*	V	Tmax blood	Tmax tissue	Pop
-	3.68	7.10	1.01	88.4	57.6	89.2	-
2s	4.51	8.95	0.16	155.3	62.3	97.1	6.0s
4s	4.61	8.56	1.05	159.4	62.4	97.1	7.2s
6s	4.63	8.54	1.06	159.5	62.4	97.1	7.9s
8s	4.56	8.37	0.80	152.9	62.2	96.1	-
10s	4.47	8.27	1.12	145.6	62.0	95.1	-

Cylindrical, Ventricle, LBF (0.1m/s), (5g,80W,6s,60mL/min)							
Interval	D*	W	DW*	V	Tmax blood	Tmax tissue	Pop
-	3.49	6.96	0.32	79.2	60.2	91.0	-
2s	4.21	8.62	0.18	130.9	65.2	97.1	4.0s
4s	4.28	8.61	0.25	134.7	65.3	97.1	4.9s
6s	4.28	8.56	0.25	133.6	65.2	97.1	5.4s
8s	4.27	8.61	0.24	132.0	65.2	97.1	5.8s
10s	4.23	8.70	0.21	128.2	65.1	96.7	-

Cylindrical, Ventricle, LBF (0.1m/s), (5g,90W,4s,60mL/min)							
Interval	D*	W	DW*	V	Tmax blood	Tmax tissue	Pop
-	3.07	6.56	0.64	60.0	61.7	87.8	-
2s	3.93	8.85	0.19	109.9	67.8	97.1	3.3s
4s	3.99	8.78	0.15	112.0	67.8	97.1	3.9s
6s	3.93	8.75	0.19	106.0	67.4	95.3	-
8s	3.84	8.55	0.19	98.9	67.0	93.6	-
10s	3.74	8.38	0.18	92.8	66.7	92.8	-

Spherical, Atrium, LBF (0.1m/s), (5g,70W,8s,60mL/min)							
Interval	D*	W	DW*	V	Tmax blood	Tmax tissue	Pop
-	3.47	6.82	1.00	75.6	61.7	88.6	-
2s	4.32	9.42	0.26	144.4	70.5	97.1	7.0s
4s	4.38	9.43	0.22	146.5	70.5	97.0	-
6s	4.31	9.37	0.25	138.5	70.1	95.6	-
8s	4.24	9.31	0.28	131.4	69.8	94.6	-
10s	4.17	9.32	0.20	125.2	69.6	93.7	-

Spherical, Atrium, LBF (0.1m/s), (5g,80W,6s,60mL/min)							
Interval	D*	W	DW*	V	Tmax blood	Tmax tissue	Pop
-	3.29	6.68	1.04	68.1	64.6	91.4	-
2s	3.88	9.55	0.13	115.3	73.0	97.1	4.1s
4s	3.95	9.51	0.19	117.6	73.0	97.1	5.0s
6s	3.96	9.50	0.20	117.5	73.1	97.1	5.6s
8s	3.92	9.51	0.18	114.6	73.0	97.1	5.9s
10s	3.86	9.53	0.13	109.9	72.9	96.4	-

Spherical, Atrium, LBF (0.1m/s), (5g,90W,4s,60mL/min)							
Interval	D*	W	DW*	V	Tmax blood	Tmax tissue	Pop
-	2.97	6.20	0.92	52.2	66.4	89.2	-
2s	3.62	9.68	0.16	95.2	75.3	97.1	3.2s
4s	3.66	9.67	0.10	96.0	75.3	97.1	3.8s
6s	3.62	9.66	0.15	91.5	75.0	96.0	-
8s	3.53	9.58	0.17	85.0	74.6	94.4	-
10s	3.45	9.51	0.19	79.8	74.3	93.2	-
