# Supplementary material for: Systematic Characterization of High-Power Short-Duration Ablation: Insight From an Advanced Virtual Model
Source: Front Med Technol. 2021 Nov 12;3:747609. doi: 10.3389/fmedt.2021.747609 (PMC8757782; doi:10.3389/fmedt.2021.747609)
Supplement: Supplementary file 3 [file Data_Sheet_3.PDF]

Supplementary Material: Systematic characterization of High-Power Short-Duration Ablation: Insight from an advanced virtual model.

Argyrios Petras [1], Zoraida Moreno Weidmann [2], Massimiliano Leoni [1], Jose M. Guerra [2,*], and Luca Gerardo-Giorda [1,3]

[1] *RICAM, Austrian Academy of Sciences, Linz, Austria*
[2] *Department of Cardiology, Hospital de la Santa Creu i Sant Pau, CIBERCV, and Universidad Autonoma de Barcelona, Barcelona, Spain*
[3] *Institute for Mathematical Methods in Medicine and Data-Based Modelling, Johannes Kepler University, Linz, Austria*

Correspondence [*]: jguerra@secardiologia.es

1 COMPUTATIONAL MODEL

We model Radio Frequency Ablation (RFA) as a multiphysics problem where an electrical problem (the source of the applied current), a thermal problem (the heating of the tissue and the blood surrounding the catheter), and a fluid dynamics problem (the blood and saline coolant motion) are tightly coupled. At a frequency of 500 kHz, and over the distance of interest, the biological medium can be considered totally resistive and the electrical problem can be set in quasi-static form, by solving a Laplace equation augmented by an integral power constraint in the case of a constant power ablation. The heat transfer in the blood and in the tissue is governed by a modified Penne's bioheat equation, with a source term derived from the solution of the electrical potential equation. The blood is modeled through the incompressible Navier-Stokes equation. Globally, the resulting RFA model is a triple-feedback system of time-dependent partial differential equations to be solved. In the following we collect the main characteristics of the model, that was introduced and validated in [1].

1.1 Geometry

A full three-dimensional computational geometry is considered that consists of a blood chamber, cardiac tissue, a board that models effects external to the system, an electrode and a thermistor, as shown in Figure S1 (left). We consider a 6-pores open-irrigated electrode of diameter $2.33\,\mathrm{mm}$ and length $3.5\,\mathrm{mm}$ with either a hemispherical or a cylindrical tip, following the design of state-of-the-art catheters typically used for RFA. The pores, of diameter $0.5\,\mathrm{mm}$, are connected to an inner channel of diameter $0.73\,\mathrm{mm}$ inside the electrode, which is connected to the catheter body and allows the saline to flow in the blood chamber. Figure S1 (right) shows the spherical and cylindrical electrode tips considered in our model.

The contact force exerted by the catheter induces a deformation to the cardiac tissue. For a given contact force F, we model this deformation by solving an axisymmetric Boussinesq problem for both a spherical and a cylindrical profile [2], and we directly impose it on the tissue geometry.

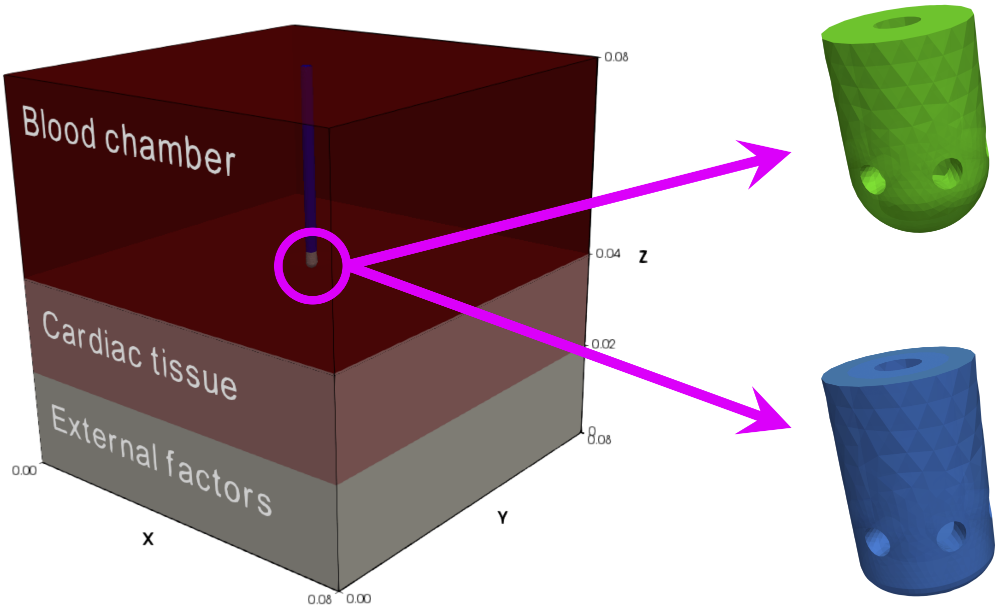

Figure S1. Left: The complete computational geometry. Right: The catheter tip designs.

1.2 Mathematical model

1.2.1 Blood and saline flow

The incompressible Navier-Stokes equations describe the blood flow and interaction of the blood and the irrigated saline from the electrode holes. Specifically:

$$\begin{aligned} \frac{\partial \vec{u}}{\partial t} + \vec{u} \cdot \nabla \vec{u} - \text{div}\, \mathbf{T}(\vec{u}, p) = \vec{0} \quad &\text{in} \;\; \Omega_{blood} \times (0, T), \\ \text{div}\, \vec{u} = 0 \quad &\text{in} \;\; \Omega_{blood} \times (0, T), \end{aligned} \tag{S1}$$

where $\vec{u}$ is the flow velocity, $\mathbf{T}(\cdot, \cdot)$ is the stress tensor, p is the pressure scaled by the density and Ω_{blood} is the blood subdomain. A constant blood inflow is considered at the plane $X = 0$ of the blood subdomain with the corresponding outflow conditions at the opposite plane. As the densities of the saline and the blood are comparable, the mixing of the two fluids can be assumed to be perfect, and the saline irrigation is modelled through the imposition of additional inflow boundary conditions at the irrigation pores: a radial inflow towards the blood is considered from the blood-saline pipes interface. No slip conditions are imposed on all other surfaces, including the internal blood-tissue interface.

1.2.2 Electrical field

The electrical potential is governed by a quasi-static equation augmented with a constraint for constant power ablation in the whole computational domain Ω:

$$\begin{cases} \text{div}\, (\sigma(T) \nabla \Phi) = 0, \\ \int_{\Omega} \sigma(T) |\nabla \Phi|^2 \, dx = P, \end{cases} \tag{S2}$$

where P is the total power dissipated in our system. A potential V_0 is considered on the catheter-electrode interface, which is calculated via optimization techniques to satisfy the power constraint equation [1]. Zero potential at the bottom of the computational domain models the dispersive electrode, while insulation boundary conditions are applied to all remaining boundaries.

1.2.3 Tissue heating

The temperature changes are modelled using a modified version of Penne's bioheat equation in the whole computational domain:

$$\rho c(T)\left(\frac{\partial T}{\partial t} + \vec{u}\cdot\nabla T\right) - \text{div}\,(k(T)\nabla T) = \sigma(T)|\nabla\Phi|^2, \tag{S3}$$

where T is the temperature, ρ is the density, c is the specific heat, k is the thermal conductivity, $\sigma(\cdot)$ is the electrical conductivity and Φ is the electrical potential. In principle, a metabolic heat gain and the heat loss due to the blood perfusion should be considered, but these terms can be omitted for short ablation time [3, 4]. Thermal insulation boundary conditions are applied on the catheter walls and the saline pipes, while a constant temperature of $22\,^\circ\mathrm{C}$ is considered on the blood-pipes interface. Body temperature of $37\,^\circ\mathrm{C}$ is applied on all the remaining boundaries.

A more detailed analysis of the mathematical model can be found in [1].

1.3 Model parameters

A number of parameters appear in the mathematical model that are drawn from the literature [5]. The specific heat c, thermal and electrical conductivities k and σ are considered temperature dependent within the tissue [6]:

$$c(T) = c_0(1 + c_1(T-37)), \qquad k(T) = k_0(1 + k_1(T-37)), \qquad \sigma(T) = \sigma_0(1 + \sigma_1(T-37)).$$

The electrical conductivity σ_b of the board underneath the tissue is tuned to match the initial resistance of the system and power delivered to the tissue, which is calculated as follows [7]

$$P_{tissue} = \frac{A_{tissue}\,\sigma_0^{(tissue)}}{A_{blood}\,\sigma_0^{(blood)} + A_{tissue}\,\sigma_0^{(tissue)}}\,P_{abl} =: \alpha P_{abl}, \tag{S4}$$

where P_{abl} is the total power set by the ablation protocol, (A_{blood}, $\sigma_0^{(blood)}$) and (A_{tissue}, $\sigma_0^{(tissue)}$) are the contact area of the electrode with the blood and the tissue respectively, along with the corresponding electrical conductivities at body temperature. The initial resistance of the system is set as 120 Ω. More details on the calculation of σ_b can be found in [1].

The model parameters are collected in Table S1, and additional details can be found also in [8].

1.4 Simulation protocol

The numerical approximation is based on finite elements. The model is implemented in a self-developed code, written in C++ and based on FeniCS-HPC (http://www.fenics-hpc.org), an open source finite element simulation platform. CFD simulations of the blood/saline flow are performed

Table S1. Model parameters.

	Blood	Tissue	Electrode	Thermistor	Board
ρ ($\mathrm{kg\,m^{-3}}$)	1050	1081	21500	32	1076
c_0 ($\mathrm{J\,kg^{-1}\,K^{-1}}$)	3617	3686	132	835	3017
c_1 (°C^{-1})	-	-0.0011	-	-	-
k_0 ($\mathrm{W\,m^{-1}\,K^{-1}}$)	0.52	0.56	71	0.038	0.518
k_1 (°C^{-1})	-	0.0022	-	-	-
σ_0 ($\mathrm{S\,m^{-1}}$)	0.748	0.381	4.6×10^6	10^{-5}	σ_b
σ_1 (°C^{-1})	-	0.015	-	-	-
ν (-)	-	0.499	-	-	-
E (kPa)	-	40	-	-	-

using a stabilized Galerkin Least Squares (GALS) Navier-Stokes solver for incompressible flows. As the thermal problem is advection-dominated in the blood domain, a SUPG (Streamline-Upwind Petrov-Galerkin) stabilization term is added to the discrete Bioheat equation. The different temporal scales of the dynamics involved prompt the use of different time steps, a coarser one for the Bioheat and a finer, adaptive one for Navier-Stokes: the models are thus synchronized at the larger time step, where also the potential equation is solved. The three dimensional mesh for the computational domain is generated with the open source software Salome [9], with about 5,000,000 tetrahedral elements of variable size (ranging from $0.01\,\mathrm{mm}$ and $1\,\mathrm{mm}$) to ensure a sufficiently accurate description of the electrode, the contact area and its neighborhood. The mesh refinement in the area of the catheter is implemented to have a better control of the fluid dynamics in that sensitive area. More details on the discretization and the numerical solution of the mathematical model can be found in [1].

1.5 Lesion assessment

Irreversible tissue damage is inflicted on the cardiac tissue at $50\,°\mathrm{C}$ [10]. The computational lesion is identified by the $50\,°\mathrm{C}$ isotherm contour in our numerical experiments using the open source software Paraview [11]. The lesion morphology is identified by its depth (D), its width (W), its depth at which the maximum width occurs (DW), and its volume (V). Depth measurements are taken from the undeformed surface as shown in Figure S2.

REFERENCES

[1] Argyrios Petras, Massimiliano Leoni, Jose M Guerra, Johan Jansson, and Luca Gerardo-Giorda. A computational model of open-irrigated radiofrequency catheter ablation accounting for mechanical properties of the cardiac tissue. *Int J Numer Meth Biomed Engng.* 2019; 35:e3232. https://doi.org/10.1002/cnm.3232

[2] Ian N Sneddon. The relation between load and penetration in the axisymmetric boussinesq problem for a punch of arbitrary profile. *International Journal of Engineering Science*, 3(1):47–57, 1965.

[3] Enrique J Berjano. Theoretical modeling for radiofrequency ablation: state-of-the-art and challenges for the future. *Biomedical Engineering Online*, 5(1):24, 2006.

[4] Ana González-Suárez, Enrique Berjano, Jose M Guerra, and Luca Gerardo-Giorda. Computational modeling of open-irrigated electrodes for radiofrequency cardiac ablation including blood motion-saline flow interaction. *PloS ONE*, 11(3):e0150356, 2016.

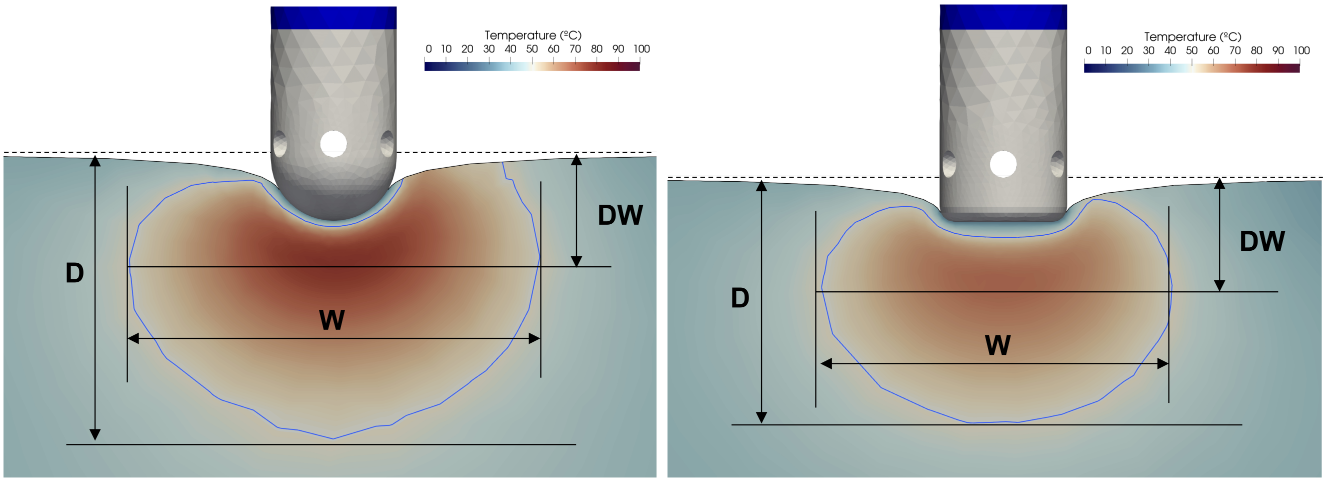

Figure S2. The indicators of the lesion size assessment.

[5] P.A. Hasgall, F. Di Gennaro, C. Baumgartner, E. Neufeld, B. Lloyd, M.C. Gosselin, D. Payne, A. Klingenböck, and N. Kuster. Itis database for thermal and electromagnetic parameters of biological tissues. *Version 4.0*, 2018.

[6] Francis A Duck. *Physical properties of tissues: a comprehensive reference book*. Academic Press, 2013.

[7] Fred HM Wittkampf and Hiroshi Nakagawa. Rf catheter ablation: Lessons on lesions. *Pacing and Clinical Electrophysiology*, 29(11):1285–1297, 2006.

[8] Argyrios Petras, Massimiliano Leoni, Guerra Ramos JM, Johan Jansson, and Luca Gerardo-Giorda. Tissue drives lesion: computational evidence of interspecies variability in cardiac radiofrequency ablation In: Coudière Y., Ozenne V., Vigmond E., Zemzemi N. (eds) *Functional Imaging and Modeling of the Heart. FIMH 2019.* Lecture Notes in Computer Science, vol 11504, pp. 139-146. Springer, Cham

[9] Andre Ribes and Christian Caremoli. Salome platform component model for numerical simulation. In *Computer Software and Applications Conference, 2007. COMPSAC 2007. 31st Annual International*, volume 2, pages 553–564. IEEE, 2007.

[10] Shoei K Stephen Huang and Mark A Wood. *Catheter Ablation of Cardiac Arrhythmias E-book*. Elsevier Health Sciences, 2014.

[11] Utkarsh Ayachit. *The paraview guide: a parallel visualization application*. Kitware, Inc., 2015.
